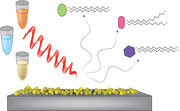# Influence of core size and capping ligand of gold nanoparticles on the desorption/ionization efficiency of small biomolecules in AP‐SALDI‐MS

**DOI:** 10.1002/ansa.202000900

**Published:** 2020-10-01

**Authors:** Zhen Liu, Peng Zhang, Andrea Pyttlik, Tobias Kraus, Dietrich A. Volmer

**Affiliations:** ^1^ Institute of Bioanalytical Chemistry Saarland University Saarbrücken Germany; ^2^ School of Materials Science and Engineering Sun Yat‐sen University Guangzhou China; ^3^ INM‐Leibniz Institute for New Materials Saarbrücken Germany; ^4^ Institute of Colloid and Interface Chemistry Saarland University Saarbrücken Germany; ^5^ Department of Chemistry Humboldt‐Universität zu Berlin Berlin Germany

**Illustrator: Adriana Savastano**

**Email**: adry.sav@hotmail.com

Gold nanoparticles (AuNP) are frequently used in surface‐assisted laser desorption/ionization mass spectrometry (SALDI‐MS) for analysis of biomolecules because they exhibit suitable thermal and chemical properties as well as strong surface plasmonic effects. Moreover, the structures of AuNP can be controlled by well‐established synthesis protocols. This was important in the present work, which studied the influence of the nanoparticles' structures on atmospheric pressure (AP)‐SALDI‐MS performance.

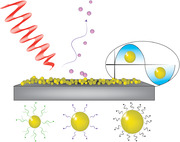

A series of AuNP with different core sizes and capping ligands were investigated, to examine the desorption/ionization efficiency (DIE) under AP‐SALDI conditions. The results showed that both the gold nanoparticles' core size as well as the nature of the surface ligand had a strong influence on DIE. DIE increased with the size of the AuNP and the hydrophobicity of the ligands. Chemical interactions between ligand and analytes also influenced DIE.

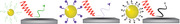

The optimized AuNP were successfully used to analyze a wide arrange of different low molecular weight biomolecules as well as a crude pig brain extract, which readily demonstrated the ability of the technique to detect a wide range of lipid species within highly complex samples.